# mHealth information for migrants: an e-health intervention for internal migrants in Vietnam

**DOI:** 10.1186/s12978-016-0172-6

**Published:** 2016-05-14

**Authors:** Lan Thi Hoang Vu, Ngan Thi Kim Nguyen, Hanh Thi Duc Tran, Nazeem Muhajarine

**Affiliations:** Department of Epidemiology and Biostatistics, Hanoi School of Public Health, Hanoi, Viet Nam; Department of Community Health and Epidemiology, University of Saskatchewan, Saskatoon, Canada

**Keywords:** Mobile health, Reproductive health, Sexual health, Intervention, Evaluation, Female migrants

## Abstract

**Background:**

Socio-economic development in Vietnam has resulted in increased internal migration particularly among young women seeking employment opportunities in cities. Vietnamese female migrants who enter new environments often encounter the loss or neglect of their right to access sexual and reproductive health services. To address this, a mobile health (mHealth) intervention model was implemented over 12 months (2013–2014) in a factory in the Long Bien industrial zone of Hanoi, Vietnam.

**Methods:**

The intervention provided sexual and reproductive health services for female migrants through text messaging, information booklets accompanied maps, and free counseling via a hotline. To evaluate the impact of the intervention, pre- and post-intervention data were collected to measure changes in women’s knowledge and practices related to sexual and reproductive health. Qualitative data in the form of personal interviews were also collected. The sample size for the baseline survey was 411 women, and for the post-intervention survey it was 482 women (the intervention involved an open cohort). The majority of women were unmarried and under the age of 25.

**Results:**

Results indicate that there was high uptake of the intervention services and that most women found the services important and useful. In addition, there was evidence that the intervention (1) increased women’s knowledge of sexual and reproductive health (e.g., proper use of condoms, identification of high-risk behaviors such as having unprotected sex), and (2) fostered improved practices related to sexual and reproductive health (e.g., increased gynecological check-ups and use of condoms).

**Conclusions:**

The study demonstrated the feasibility of implementing a multi-faceted intervention for migrant women working in an industrial zone in Hanoi, Vietnam as well as its successful uptake and some early positive effects. This can be used to inform future design and implementation of mHealth/eHealth intervention models for migrant and other vulnerable/hard to reach population.

## Background

Socio-economic development resulting from the *DoiMoi* reforms in Vietnam has resulted in increased internal migration as people have and continue to move away from their communities of origin in search of economic opportunities [1, 2]. Women increasingly predominate in the internal migrant population; most of them are young [3, 4]. A concern regarding migrants in Asia, including in Vietnam, is with respect to the loss or neglect of their rights in their new environments, including rights to access reproductive health information and services. Barriers to accessing services are frequently institutional, exposing migrants to greater risk of illness than non-migrant people and often excluding them from formal medical systems [5]. At the same time, socio-cultural factors play a part in influencing the behavior of migrant women, as well as their access to health services [6].

With funding from Grand Challenges Canada, a mobile health (mHealth) intervention model for internal female migrants was implemented over 12 months (2013–2014) in a factory in the Long Bien industrial zone of Hanoi, Vietnam. The project focused on a target population in great need of a health intervention in particular in the sexual and reproductive health area. Female migrants, who are doubly vulnerable because they are female and are migrants, typically have little control over the choices available to them or the power to avoid high-risk behaviours. A focus on sexual and reproductive health is relevant because the majority of female migrants are young, sexually active, and is likely to be less informed about sexually transmitted diseases (STDs) and HIV and/or less likely to engage in safe sexual practices [7]. This mHealth model provided reliable, low-cost, timely and customized reproductive health advice for migrant women workers via text messaging, booklets, maps, and free counseling via a hotline. This paper aims to (1) describe the implementation of the project, and (2) measure the impact of the mHealth intervention on female migrants in Vietnam by tracking changes in their knowledge and practices related to sexual and reproductive health.

## Methods

### Intervention setting

The intervention was conducted in the Long Bien district of Hanoi, the political capital and one of the two economic and cultural centers in Vietnam. Long Bien is a rapidly growing district, with national highways intersecting and new urban compounds and industrial zones such as Sai Dong A and Sai Dong B located within. These changes have contributed to increased inflow of rural-to-urban migrants in Long Bien. To date, migrant populations accounts for 10 % of the population in Long Bien. Most migrants work in the industrial zones and in private small enterprises (PSE) [8].The X Company is a state-owned enterprise under the Hanoi Service of Industry. It is located in the Sai Dong B industrial zones in Long Bien and produces metals for export and domestic companies. This company was chosen as target population for the intervention due to their high number of young female migrant workers.

### Study design

This study applied a pre-experimental design with pre- and post-intervention measures. A mixed-methods approach was employed, using both quantitative and qualitative methods to collect in-depth information about the intervention’s progress and impacts. The criteria for selecting subjects were: female migrant workers aged 18–49 years, and have a permanent contract with the company for at least 12 months during the intervention period.The sample size for the baseline survey was 411 women, and for the post-intervention survey it was 482 women (as the intervention population was an open cohort, women kept joining the study during the implementation phase). The intervention period was 12 months, from June 2013 to June 2014. The baseline survey was done in April 2013 and the post-intervention survey was done in June 2014. The sample size for both surveys included all women working in the factory satisfied the above mentioned criteria. As the study design was pre-experimental design, efforts was taken to control for co-intervention bias and loss to follow up bias. For co-intervention bias, as we don’t have control group, the effect observed in this study may due partially/totally to other intervention programs implemented at the same time with our program. To control for this bias, we had chosen a target population without any other intervention program (as stated in the annual health care program); worked closely with the health department in the factory to collect information about other interventions but during the intervention time, to our knowledge there was no other intervention activities in the field of our program. Loss to follow up was not a problem in our study as the study time only 12 months, subjects selected had permanent contract with factory so we have more subjects involving in study, no one withdrew.

### Quantitative/qualitative data collected in the survey

Table 1 presents the data collected in the survey.

**Table 1:** Quantitative/qualitative indicators collected in the study

Type of services	Quantitative indicators (Survey of female workers)	Qualitative indicators
Hotline	• Frequency of services • Ranking frequent topic of call • Number of calls over time • % of women ever heard about the services • % of women ever used services • Ranking about quality/importance of services	• In-depth information about issues when implementing services from service provider (hotline expert) • In-depth information about relevance, quality and usefulness of services from female workers
Map of health care services providers	• Number of health services provided surveyed and presented in map • % of women ever received map • % of women ever used map to find health care providers • Ranking about quality/importance of services	• In-depth information about relevance, quality and usefulness of services from female workers
SMS	• Frequency of services • Number of SMS developed • Number of SMS sent over time • Major topic of SMS sent • % of women ever heard about the services • % of women ever used services • Ranking about quality/importance of services	• In-depth information about relevance, quality and usefulness of services from female workers

### Description of intervention

The intervention provided the following services: hotline, sending communications via short message service (SMS), and a map of health services providers. For each service, information was collected in relation to its use, relevance, quality, and perceived usefulness. In order to evaluate the impact of the intervention, information about changes in female migrants’ knowledge and practices relating to sexual and reproductive health was collected. Changes in knowledge were measured in the following areas: sexually transmitted diseases (STDs; names, symptoms, means of transmission), human immunodeficiency virus (HIV) and acquired immunodeficiency syndrome (AIDS), reproductive tract infections (RTIs), contraceptive methods, and abortion. Changes in practices relating to sexual and reproductive health were assessed through the percentage of (1) unmarried participants who reported using a condom in their last sexual intercourse, and (2) married participants who reported having a gynecological check-up during the last six months; these are important indicators for accessing reproductive health services in Vietnam and were identified as priority needs of target population at baseline survey.

### Ethical approval

The study protocol was approved by the Ethical Clearance Committee at Hanoi School of Public Health. All the subjects were fully explained about the objectives/process of the study and signed consent forms before participating the study.

## Results

### Characteristics of intervention cohort

The inception cohort consisted of 411 female migrants; in addition, 71 participants joined the open cohort, for a total final sample size of 482. The latter participants were introduced to the project either by their friends who already registered in the system or by staff in the factory health care center. Table 2 presents the demographic characteristics of the inception cohort. The intervention sample was quite young (mean age 21.6 years) and predominantly unmarried (84 %). The majority of respondents (61 %) reported that their highest level of education completed was high school or greater, although a sizeable minority (39 %) had completed less than high school. Just under two-thirds of the women had only migrated once, and similar proportion (64 %) had health insurance; almost all were registered temporary residents.

**Table 2:** Demographics of intervention group at baseline survey (*n* = 411)

Variable	*n*	%
Average age (years)	21.6 ± 3.2	
Average income (in millions of VND)	1.3 ± 1.2	
(1 USD = 22,500 VND)		
Average temporary residence period at Long Bien(months)	5.0 ± 3.8	
Marital status		
*Unmarried*	345	83.9
*Married*	66	16.1
Highest education		
*Primary*	25	6.1
*Secondary*	136	33.1
*High school*	175	42.6
*Vocational school*	27	6.6
*College/university and higher*	48	11.7
Number of migrations		
*1 time (the first time)*	260	64.2
*Many times (with specific number)*	40	9.9
*Many times (without number)*	81	20.0
*Not sure*	24	5.9
Registered temporary residence	405	98.5
Have health insurance	263	64.0

### Intervention activities

#### Hotline services

A 24/7 (i.e. opened 24 hours a day, 7 days a week) free, secure and confidential hotline operated by reproductive health specialists was open to clients for consultation about reproductive health and family health. A total of 768 calls were made to the hotline, of which 518 calls (67.4 %) were from system registered clients (i.e. people who registered for the MSM services) and 250 calls (32.6 %) were from new clients. The average number of calls per client was about two. The maximum re-call number was four times. Each consultation took an average of five minutes. The longest consultation took about 10 minutes, whereas the shortest one took around two minutes.

**Fig. 1:**
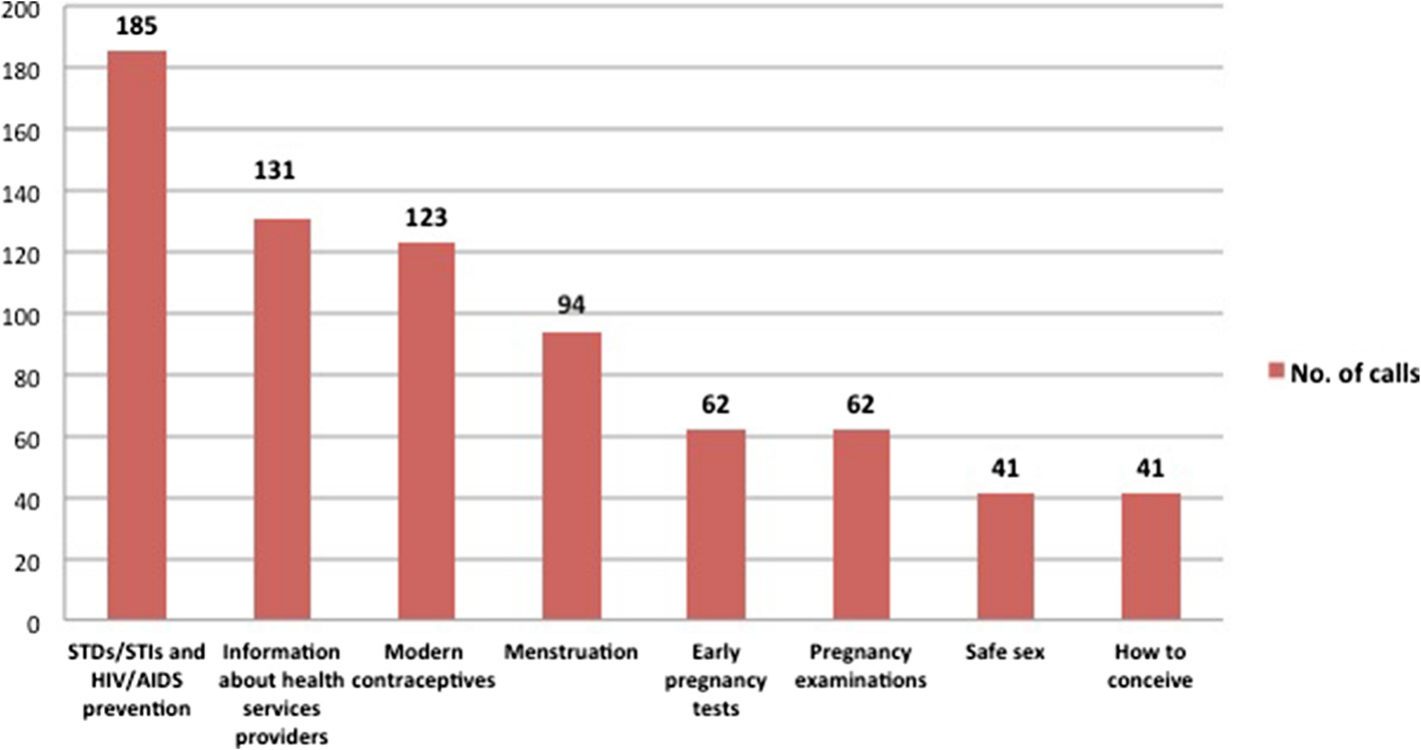
Main hotline topics

The main topics of hotline consultations were STDs/sexually transmitted infections (STIs) and HIV/AIDS prevention, modern contraceptives, information about health services providers, and menstruation issues (see Fig. 1).

The interview with the specialized doctor in charge of the hotline revealed important details about this service. First, most of the calls to the hotline were made after regular office hours. This is a key factor because it shows that migrants often need services after working hours; however, all government health services currently operate only during regular business hours. Therefore, if this service did not exist, women would have looked for care from sources such as private clinics, which typically have cost-barriers. Second, the most frequently asked questions were about sex and STDs, topics considered taboo in Vietnamese culture. Because the risks for STIs and HIV are elevated with high-risk behaviours (e.g., sexual intercourse with multiple partners) and sexual prejudice still exists in Vietnam, women who are at risk for sexually transmitted diseases are often reluctant to go for a check-up or treatment in a timely manner [6]. For female migrants, the fear of judgement and social exclusion is even worse due to their relatively small and homogeneous social network, which is typically linked to their home villages. Since the hotline service is anonymous, it helped to eliminate their fear and reluctance to seek information. Third, as a point of reflection and learning, in the future, interventions such as this should clearly outline the methods employed to ensure users’ confidentiality. The most significant barrier for clients to use this service was their concern about the confidentiality of their information.

There were additional insights gained through interviews conducted on the use and effectiveness of the hotline service. For example, commenting on the easy accessibility of the service, one client said: *“We had long working hours, once we were off, all the government health services had already closed; if we need to use health care, we have to use private services, which are much more costly, and we will not seek help immediately. The hotline is 24/7, it is so easy for us to use. In the future, even if we have to pay fee for that, if it is not too expensive, I will still use it because it is so easy and be there for me when I need it”*

In Vietnam attitudes about sex are changing, and there is a new openness to the topic. Information about sex and sexuality is becoming popular in the media, newspapers, television shows, websites and books. However, among some groups, such as women, people of certain religions and cultural backgrounds, people living in rural areas, there remains a taboo on openly discussing sexual matters. Given this, commenting on the private and confidential nature of hotline services, one 20 year old participating woman said *“Vietnamese women are still expected to be sexually innocent until marriage. There are many questions we cannot ask our friends or our family about contraceptives or sex. This service give me a great opportunity to ask many questions I’ve never be able to ask before.”*

However, the participants also raised some limitations of the intervention such as the implementation was limited to only one year and only one hotline expert was available:

A female 19 year old stated *“I wish the project can continue for longer time, you see, the hotline is closed now and when I need information I don’t have anyone to ask. Also in the factory, each year there will be many new workers, they won’t be able to receive SMS for hotline service”.*

#### SMS services

In total, 170 specific SMS messages were developed by a health expert, covering seven sexual and reproductive health topics. These topics were developed based on results from the needs assessment survey [9]. Each SMS message was no longer than 160 characters (maximum allowable characters). An example includes: *“Symptoms of STDs: discharge, yellowish or greenish vaginal discharge, genital itching or irritation, pain during sexual intercourse.”*

Generally, SMS messages were sent about three times per week after working hours (by 5 pm) to clients who registered to receive them. Among the 444 women who had ever received an SMS message, most of them found the SMS service and content useful or very useful (85 % and 88 %, respectively; see Table 3). All the women think that the service is important for female migrants, and almost all (97 %) said that they would like to receive more SMS messages in the future.

**Table 3:** Ratings of SMS service and content

	*n* (out of 444)	*%*
*SMS service*		
Not very useful	6	1.4
Not useful	18	4.1
Neutral	42	9.5
Useful	288	64.9
Very useful	90	20.3
*Content of SMS messages*		
Not useful	19	4.1
Neutral	36	8.1
Useful	216	48.6
Very useful	174	39.2

The content of the SMS was easy to understand. In all of the interviews and group discussionsit was mentioned that the SMS included simple language with specific guidance about what the participants need to know or do, and on a given week these messages covered a given topic. For instance, a female 23 year old mentioned “SMS was good, each SMS was short but informative, and project always sent related SMS within a week or two. In a week, we received 3 SMS about different ways to detect pregnancy, in another week, we received 3 SMS about STDs.”

One limitation of this service relates to the need to re-register the phone number when a participant changes her phone number. An interview with a 20 year old female migrant worker stated “I had to register for the program twice because I changed phone number, you know, we changed our phone quite frequently every 3 or 6 months when we found good promotion program from phone service. It was easy to register again but still, sometime you may forgot to register for a while when you change your phone.”

#### Map of local health services providers

**Fig. 2:**
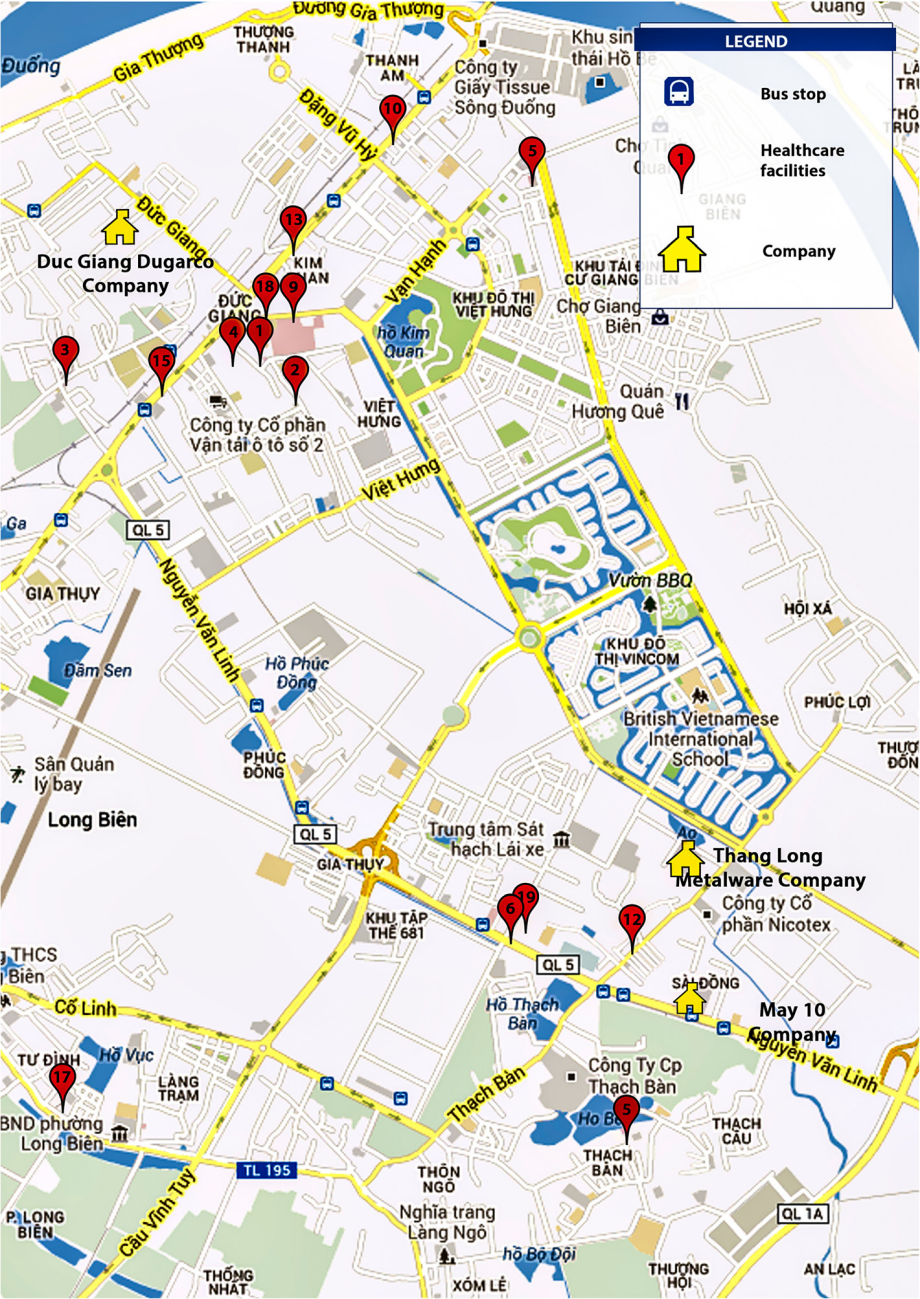
Sample map of local health services providers

A map of reproductive health care providers was developed and included summary information (services offered, address, hours of operation, equipment, staff credentials, insurance, average cost, and promotion). The map contained profiles and locations of 19 reproductive health care facilities and 186 pharmacies (see Fig. 2). All participants received a map and a small booklet of essential information on reproductive health. When asked about the most useful topics in the booklet and map, cost of services (31 %), location of health care centers (26 %), and contraceptive methods (22 %) were mentioned most often. Most women (84 %) agreed that the booklet provided detailed information about all reproductive health topics that were of significance to them. In general, women found the booklet was useful for finding local health care centers.

### Impact of the mHealth intervention on female migrants

#### Changes in knowledge

Post intervention, compared to pre-intervention, women demonstrated significantly more knowledge pertaining to STDs (e.g., names, symptoms, and means of transmission; see Table 4). In addition, more women correctly identified the risk of unprotected sex and the importance of assessing risk of sexual partners who may have STDs. As with knowledge of STDs, it was observed that women knew more about HIV and AIDS following the intervention compared to at the baseline (e.g., they could better identify high-risk behaviors such as having sex without a condom). Although there still existing misconceptions about how HIV/AIDS can be transmitted, there was a significant increase in women’s knowledge of HIV prevention methods. Most were also able to list locations offering HIV testing and counseling. Post-intervention, women were more likely to understand symptoms and high-risk behaviors associated with RTIs.

**Table 4:** Changes in knowledge of STDs

	Before (*n* = 411)	After (*n* = 411)	*p*-value
*Names of STDs*			
*Never heard of STDs*	64 (15.6)	51 (12.4)	0.21
*List 1 disease*	169 (41.1)	99 (24.1)	<0.001
*List at least 2 diseases*	178 (43.3)	261 (63.5)	<0.001
*High-risk behaviors for HIV*			
*Do not keep genitals clean (False)*	64 (15.6)	62 (15.1)	0.92
*Having sex with multiple partners without a condom (True)*	251 (61.1)	302 (73.5)	<0.001
*Having sexual intercourse with someone who has HIV, without a condom (True)*	356 (86.6)	379 (92.2)	0.01
*Sharing needles (True)*	286 (69.6)	318 (77.4)	0.02
*Kissing (false)*	27 (6.6)	21 (5.1)	0.46
*HIV prevention methods*			
*Abstinence*	79 (19.2)	169 (41.1)	<0.001
*Condom use*	371 (90.3)	400 (97.3)	<0.001
*Sex with just one partner*	138 (33.6)	215 (52.3)	<0.001
*Avoid sex with commercial sex workers*	241 (58.6)	375 (91.2)	<0.001
Names of contraceptive methods			
*List at least 3 methods*	321 (78.1)	354 (86.1)	0.003
*List 1 or 2 methods*	75 (18.2)	47 (11.4)	0.009
*Don’t know*	15 (3.6)	10 (2.4)	0.41

Respondents were asked to state all contraceptive methods, at post intervention. Eighty-six percent of women, as compared to 78 % pre-intervention, listed at least three methods correctly. Condom use was still the most common method stated (96 %). There was only 5 % women who know about cervical cap; this is explained by the fact that cervical cap is one of the least common method of contraception in Vietnam.

When asked how to use condom correctly, a much higher proportion of women had higher scores after the intervention (63 % vs. 37 % before intervention). To receive a high score on knowledge of condom use effectively, respondents had to have at least 4 out of 7 correct optional answers as well as no incorrect answers. Although a large proportion of respondents knew that it is important to use a new condom in every sexual intercourse (80 %), or check condom for damage before use (73 %), many respondent still could not able to tell the right way to use a condom, particularly: 77 % did not squeeze the tip of condom to remove air, 50 % did not grasp the bottom of condom to withdraw, nearly 40 % did not know they have to use the condom from start to finish and over 30 % did not check the expired date before use.

#### Changes in practices of reproductive health

Among unmarried women (*n* = 345), in the baseline survey, 34 women reported that they have ever had sex (9,8 %); of those who reported having had sex, 8 women (23.5 %) reported using condom during their last sexual intercourse. In the post intervention survey, due to the addition of new participants, there were 411 unmarried women, and among them 45 women reported that they have ever had sex (10.9 %); of those who reported having had sex, 16 women (35,5 %) reported using condom during their last sexual intercourse.

Although the prevalence of using condom among unmarried women increased, due to the small sample size of those reported having had sex, the Chi-square test was not significant. Among married women in the study, the prevalence of having gynecological consultations during the last 6 months increased from 7.6 % at the baseline survey to 14.1 % in the post intervention survey, but this change was not statistically significant.

Apart from the direct impacts on female migrants, this project had created a valuable working partnership among project implementer, local authorities, factory owner, health service providers and users.One local authority officer stated “*This project was very good because it informed the local authority about project activities since the beginning of the project; it also worked closely with factory owners and health care providers in the local areas. The migrant population in our area is increasing overtime and we really need to have health programs to help them but we don’t want programs not registering with the local authorities; we want to be aware of all intervention programs in our area to avoid overlaps in service providers and interest conflicts between services providers as well to ensure benefit for service users.*”

In addition, a deputy director of the factory said *“We appreciated the services provided by the project, the project fully informed us about all of their activities and asked for our collaboration during the time they implemented. Their activities were harmonized with our factory working agenda. This was the first project that I saw where there was a strong collaboration between our factory and the project.”*

All stakeholders offered strong support to the project activities because they were able to clearly observe the benefits that accrued from the project. It is commonly known that factory owners and employers do not welcome project activities in their factories, especially during work hours. However, they also would need to keep their workers healthy in order to achieve good productivity. The interventions of this project had direct benefits to the factory workers who participated, without it interfering in their work schedule. The head of the health care center in the factory stated *“By receiving your SMS [an intervention component], the knowledge and practices toward reproductive health of our workers improved. I can observe that through contacting with our workers from time to time. They also become more open when asking for contraceptive methods such as condoms from our center.”*

For health care service providers in the local area, the project is a channel for them to promote their services to many potential users. As one physician working in a private clinic said, *“Good, I see the project map and introduction about our services, very impressive. Recently, from time to time, I had new clients from industrial factories so I guess they used the map to find our services.”*For the local authorities, it added valuable services to the local effort to support migrant population. Currently, the local authorities did not have much funding to support migrant population but they need to set up a system to help migrants, since migrant workforce is a valuable resource for local economic development. Interview with the head of the district health center showed that *“We also used the materials produced by your project, the booklet and the map to distribute to our clients, these materials are very useful”*

## Discussion

A powerful driver and consequence of modern economic development in this globalized era is the unremitting migration of people from rural villages to cities and commercial hubs seeking opportunities for work. The internal migration patterns in Vietnam are no different. The internal migrant population in Vietnam, especially female migrants, has been growing significantly over time, but not much effort has been directed toward meeting their health care needs. Vietnam’s legal framework and enforcement capacities have not kept up with these developments, to ensure the protection of people who choose to migrate internally, especially women [8]. This project was one of the first efforts known to us, to systematically work with local authorities, factory owners and health care providers in Vietnam to provide a suite of interventions centred on mobile health information services to female migrant population.

The results of this intervention study indicate that there was high uptake of the various intervention services by the women who were enrolled to receive these services, and that most women found the services timely and helpful. In addition, specifically, there was evidence that the intervention, (1) increased women’s knowledge of sexual and reproductive health, and (2) fostered better practices related to sexual and reproductive health.

Previous studies on reproductive health for migrant workers have shown that it can be difficult to get long-term cooperation from employers of these migrant workers [10], as they do not welcome activities in their factories, especially during work hours. However, this intervention addressed this concern through an innovative approach by using SMS and hotline. The SMS service was low cost, convenient way to harness the increasing ownership of mobile phones among populations such as migrant women. The required brevity of these messages also ensured that the information was clearly and simply communicated so that it could be easily understood and remembered. These messages could also be passed on from one client to her network of friends, so that they could also benefit from the information. The counseling hotline was a way to give personal health advice on demand and answer the migrants’ specific questions. The project provided a 24/7, free, confidential hotline operated by reproductive health specialists, open to any questions on reproductive and family health matters for the clients that called in. Thanks to the specificity of the hotline consultation, the experts were also able to recommend health care service locations in a vicinity convenient to the client. As most calls were made after working hours, with some very late at night, the hotline addressed the need many migrants had for time sensitive advice or urgent advice. The findings of this study suggest that the services associated with this intervention brought benefits to their workers without interfering with their work schedules.

The study revealed, however, a few shortcomings of the intervention. First, due to funding constraints, the project was only in operation for one year. Most interviewees said that the project was too short and that there was need to extend the project for a longer period of time. Second, a need was identified for similar interventions for other migrant populations in the area. Apart from migrants working in the industrial zones, there are also self-employed or entrepreneurial migrant workers, including seasonal migrants or those who work as domestic helpers. Due to their temporary civil registration status, these migrants do not have health insurance coverage in the city, because whatever policies they might have had in their place of origin are not transferrable in their relocated cities and regions. Only migrants with a formal employment contract in industrial zones receive reproductive health care through the formal health system, because they are covered by health insurance included in their contract. Thus, self-employed migrant workers are in an even more vulnerable situation than migrants working in industrial zones, since they often do not have health insurance and are not familiar with the health care system in the city. Third, it was felt that the intervention needs to extend to other topics. The interviews revealed a perception that, in addition to sexual and reproductive health, migrants would benefit from information on mental health, alcohol, tobacco and substance use and control, healthy diet, and emerging/re-emerging diseases if and when outbreaks happen. Finally, security and privacy concerns were noted. For mHealth to succeed, cooperation between local communities and regional and national health information systems is essential, because any project using mobile technologies in Vietnam needs to comply with national privacy laws. In addition, for the service users, the project needs to make confidentiality measures clear (e.g., secure storage of personal information such as phone numbers, non-sharing of these information).

## Conclusion

The finding from this study generalizes and informs future design and implementation of mHealth/eHealth intervention models particularly for migrant and vulnerable women working in large cities and regions in Vietnam and beyond. We have demonstrated the feasibility of implementing a multi-faceted intervention for migrant women working in an industrial zone in Hanoi, Vietnam, and moreover it’s successful uptake and some early positive effects.
